# Strength and muscle mass development after a resistance-training period at terrestrial and normobaric intermittent hypoxia

**DOI:** 10.1007/s00424-024-02978-1

**Published:** 2024-06-25

**Authors:** C. Benavente, P. Padial, B. R. Scott, F. Almeida, G. Olcina, S. Pérez-Regalado, B. Feriche

**Affiliations:** 1 Department of Physical Education and Sport, Faculty of Sport Sciences, University of Granada, Granada, Spain; 2 Centre for Healthy Ageing, Murdoch University, Perth, Australia; 3 PHysical Activity, Sport and Exercise (PHASE) Research Group, School of Allied Health (Exercise Science), Murdoch University, Perth, Australia; 4 Faculty of Sport Sciences, University of Extremadura, Cáceres, Spain

**Keywords:** Hypobaric hypoxia, Biomarkers, Performance, Strength training

## Abstract

This study investigated the effect of a resistance training (R_T_) period at terrestrial (HH) and normobaric hypoxia (NH) on both muscle hypertrophy and maximal strength development with respect to the same training in normoxia (N). Thirty-three strength-trained males were assigned to N (FiO_2_ = 20.9%), HH (2,320 m asl) or NH (FiO_2_ = 15.9%). The participants completed an 8-week R_T_ program (3 sessions/week) of a full body routine. Muscle thickness of the lower limb and 1RM in back squat were assessed before and after the training program. Blood markers of stress, inflammation (IL-6) and muscle growth (% active mTOR, myostatin and miRNA-206) were measured before and after the first and last session of the program. Findings revealed all groups improved 1RM, though this was most enhanced by R_T_ in NH (*p* = 0.026). According to the moderate to large excess of the exercise-induced stress response (lactate and Ca^2+^) in HH and N, results only displayed increases in muscle thickness in these two conditions over NH (ES > 1.22). Compared with the rest of the environmental conditions, small to large increments in % active mTOR were only found in HH, and IL-6, myostatin and miR-206 in NH throughout the training period. In conclusion, the results do not support the expected additional benefit of R_T_ under hypoxia compared to N on muscle growth, although it seems to favour gains in strength. The greater muscle growth achieved in HH over NH confirms the impact of the type of hypoxia on the outcomes.

## Introduction

Resistance training under hypoxic conditions (RTH) has become a topic of great interest for athletes, coaches, and scientists as a potential strategy to improve sports performance efficiently [28, 34, 36]. Its effects on muscular adaptations (muscle mass and strength) have been recently studied in detail [7, 15, 19, 23, 43]. However, discrepancies in the available studies among training methodologies make it difficult to draw firm conclusions about the added benefit of RTH compared to equivalent resistance training under normoxic conditions (RTN).

Current RTH literature has only reported on the results of interventions conducted in normobaric hypoxia (NH). However, data suggest that exposure to terrestrial altitude induces different and more severe physiological responses than NH due to factors related to the barometric pressure and/or partial pressure of O_2_ [38]_._ Therefore, the combination of strength exercise and hypoxic conditions could conceivably produce a potential increase in the production of metabolites (lactate, calcium or inorganic phosphate) that mediate hypertrophy mechanisms such as the elevation in systemic hormonal production, cell swelling and alteration in local myokines (IL-6, IL-10 or myostatin), among others, whose adaptations could be more effective at terrestrial altitude than the ones induced by NH [49].

Other agents, such as miRNAs, post-transcriptional regulators of gene expression, have recently been shown to play an important regulatory role in the response and adaptation to training. Specifically, some miRNAs (miR-1, -21, -23a, -29, -31, -126, -133a/b, -181, -206, -378, -486 and -696) seem to regulate important biological processes in muscle including growth, development, metabolic adaptation, and repair [16], by interacting with components of specific signaling pathways. Although their levels have been detected in several biofluids, such as plasma/serum and liquid biopsy, their stability in blood has allowed miRNAs to be considered as promising biomarkers [46]. Particularly, miR-206 is a skeletal-muscle-expressed miRNA and an IGF1/PI3K/AKT/mTOR signaling pathway target gene. It plays a key role in myogenesis during muscle cell differentiation [27], acting as a positive regulator of muscle growth [50]. Despite the lack of available information about the role of hypoxia on muscle development signaling pathways, it is supported that a combination of resistance training (R_T_) and environmental hypoxia may initiate transcriptional regulations that could potentially translate into satellite cell incorporation and higher force production in the long term [24].

In addition, there is growing evidence suggesting that the main anabolic effects of growth hormone (GH) are believed to be indirect via the conversion of GH to insulin-like growth factor-1 (IGF-1) in the liver, triggering the IGF-1-Akt-mTOR pathway [48, 50]. However, even though some studies have found a correlation between GH and/or IGF-1 increase after R_T_ and muscle hypertrophy [37], other studies failed to support this idea [22, 40]. The role of GH and IGF-1 in muscle strength and size adaptation to RTH are inconclusive and exhibit both positive [32, 60] and negative [12, 29] results. Although the hypertrophic effects of GH and IGF-1 are not well known, some studies postulate they are, in fact, additive [55].

Therefore, the primary aim of this study was to examine changes in muscle strength and size following an 8-week R_T_ program in terrestrial (hypobaric hypoxia) versus normobaric hypoxia and to compare them to the same training in normoxia. The secondary aim of this study was to quantify acute biomarkers associated with muscular development and metabolic demands during these training conditions to mechanistically explain between-condition differences in muscle strength and size changes. We hypothesize that the training period in hypoxia will increase muscle hypertrophy and maximal strength more than in normoxia. Also, the type of hypoxia will affect the magnitude of these changes, which will impact the metabolic stress and the corresponding myogenesis marker responses.

## Materials and methods

### Experimental approach to the problem

**Fig. 1:**
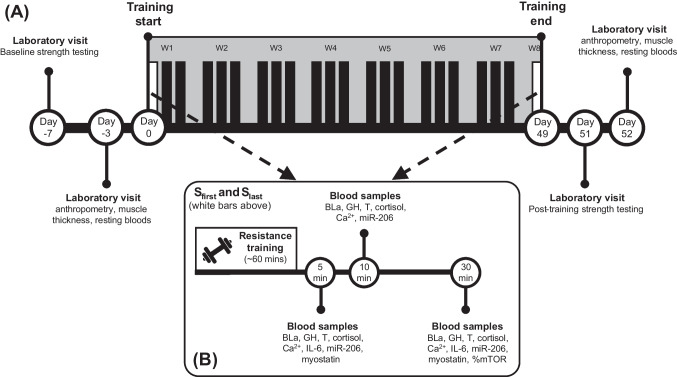
Schematic overview of the measurements obtained before and after the intervention across all groups (**A**), and the timing of biomarkers obtained following the first and last training sessions (**B**). W1 to W8: week 1 to week 8; S_first_: first session of the training program; S_last_: last session of the training program; BLa: blood lactate; GH: growth hormone; T: testosterone; Ca^2+^: calcium; IL-6: interleukin 6; miR-206: microRNA-206; % active mTOR: % active of the mammalian target of rapamycin

A longitudinal design with inter- and intra-group measurements was employed to analyze the influence of the type of moderate hypoxia (terrestrial vs normobaric) on strength, muscle mass and related serum biomarkers response after an 8-week R_T_ program (22 sessions) with respect to the same training in a normoxia condition. The participants were assigned to normoxia (N; FiO_2_ = 20.9%; ~ 760 mmHg), hypobaric hypoxia (HH; 2,320 m asl; ~ 570 mmHg) or normobaric hypoxia (NH; FiO_2_ = 15.9%; ~ 760 mmHg) for convenience. All participants lived permanently under normoxia conditions. The week before starting the intervention, the participants visited the laboratory for baseline strength testing to determine the loads used for training. Seventy-two hours before and after the study, and after 48 h of rest, the participants were measured for height (Seca 202, Seca Ltd., Hamburg, Germany), body mass (Tanita TBC-300, Tokyo, Japan) and quadriceps muscle thickness, and resting blood samples were obtained. In addition, blood samples were taken throughout the initial 30 min after the first (S_first_) and the last (S_last_) R_T_ session of the program. An overview of the design is displayed in Fig. 1.

All participants agreed to adhere to the prescribed R_T_ during the 8 weeks of the program, with no intense exercise performed other than prescribed. The participants were instructed to maintain their habits and regular dietary consumption during the entire measurement and training phases. They were provided with a protein shake supplement (111 kcal per serving; brand/product details) immediately after each session to ensure standardized nutritional intake.

All the sessions were conducted at the same time of day under the conditions of ~ 22 °C, ~ 60% humidity and < 1100 ppm CO_2_ in N; ~ 22 ºC, ~ 28% humidity and < 1100 ppm CO_2_ in HH; and ~ 23 °C, 60–90% humidity and ~ from 1500 to 6300 ppm CO_2_ in NH. The hypoxic environmental conditions were assessed by arterial oxygen saturation 5 min after the exposure to the assigned environmental condition (SpO_2_; Wristox 3100; Nonin, Plymouth, MN, USA). The participants mean resting SpO_2_ value equated to (97.2 ± 1.3; 93.9 ± 1.6 and 94.8 ± 2.2%) for S_first_ and (98.1 ± 1.5; 94.6 ± 1.3 and 94.7 ± 1.9%) for S_last_, in N, HH and NH, respectively.

### Participants

Thirty-three strength-trained males participated in the study. The participants were assigned to one of the 3 training groups: the NR_T_ group lived and trained in N (*n* = 10, age: 22.7 ± 3.4 years; height: 175.3 ± 4.1 cm; body mass: 72.0 ± 7.2 kg); the HHR_T_ group lived in N and trained in HH (*n* = 10, age: 22.8 ± 4.2 years; height: 177.5 ± 7.4 cm; body mass: 74.0 ± 13.9 kg); and the NHR_T_ group lived in N and trained in NH (*n* = 13, age: 21.9 ± 2.2 years; height: 176.5 ± 7.4 cm; body mass: 75.0 ± 8.9 kg). All participants had participated in a R_T_ regime for a minimum of 3 times per week for at least the previous 2 years. The participants were healthy with no muscular disorders and reported not taking any performance enhancing or anabolic agents during the previous month. All participants were sea-level residents and had not been exposed to an altitude or hypoxia environment of more than 1500 m asl within the 2 months before the study. This study was approved by the Local Research Ethics Committee (PEIBA/2018) and conducted following the Helsinki Declaration. Informed written consent was obtained from all participants before beginning the study.

#### Hypoxic exposure

The HHR_T_ group performed the training sessions under terrestrial hypobaric hypoxic conditions at the High-Performance Center of Sierra Nevada (2320 m asl., Spain). On every training day, participants travelled by car to the altitude center. Arrivals occurred approximately half an hour before the training session started and they immediately returned to normoxia after completing it. The NHR_T_ performed the training under simulated hypoxia in a normobaric tent (CAT 310, Colorado Altitude Training, Lafayette, CO, USA, 2.18 × 2.89 × 1.82 m). Two participants trained in the tent at the same time. The hypoxic generator system pumped the air through a semi-permeable filtration membrane (nitrogen filter technique; CAT 12, Colorado Altitude Training, Lafayette, CO, USA, 100 L/min), depleting the oxygen content until reaching a FiO_2_ = 15.9%, according to the manufacturer guidelines to equate an altitude of 2320 m. Ambient O_2_ was continuously monitored by a digital controller (Handi + , Maxtec, Salt Lake City, Utah, USA) to maintain the hypoxic conditions in the tent. Consistent with conventional routine [9, 21] NHR_T_ participants entered the tent and sat for 5 min to adapt to the training environment before starting their warm-up.

#### Resistance training program

The experimental procedure was detailed in a previous study [45]. Briefly, participants joined in an 8-week R_T_ program with 3 sessions per week performed on non-consecutive days plus an extra rest day at the end of the week. Training sessions comprised a full-body routine of 6 exercises, each performed for 3 sets of 6–12 repetitions [56], with a load ranging from 65 to 80% of 1-repetition maximum (RM) and 90 s of rest between sets and exercises [57]. The training load and volume fluctuated throughout the week (i.e., 10 repetitions per set at 70% 1RM was used in each first session of each week, 6 repetitions per set at 80%1RM in the second and 12 repetitions per set at 65% 1RM in the third). The load was individually adjusted by ~ 5% when participants exceeded the target repetition range while using a proper technique in accordance with conventional methods [1]. All routines were directly supervised by the same experts to guarantee proper technique and safe execution. Consistent encouragement was provided for participants across all training sessions to motivate them.

#### One-repetition maximum

Each participant’s 1RM was calculated for each of the six main exercises according to the National Strength and Conditioning Association guidelines [2]. Prior to the testing, participants performed a warm-up consisting of light cardiovascular exercise lasting 5–10 min followed by a set of 5 repetitions at ~ 50% of their estimated 1RM and after, 1–2 sets more of 2–3 repetitions at a load corresponding to ~ 60–80% of the estimated 1RM for the exercise. Three sets of 3–6 repetitions at increasing loads were completed before performing 1 set of 2–3 repetitions to failure. The 2-3RM load was used for 1RM estimation from the validated Brzycki’s equation [10]. Between each successive attempt, participants rested for 5 min. All 1RM determinations were made within 3 attempts. After the R_T_ program, participants repeated the same 1RM assessment for the back squat as an indicator of strength improvement from the interventions.

#### Muscle thickness

Individual muscle thickness of the quadriceps on the dominant leg was measured using ultrasound equipment (GE-LOGICQ-E portable model; GE Healthcare, Little Chalfont, UK) before and after the training period. The quadriceps were chosen for muscle thickness assessment as they are prime movers in the squat exercise, which was used to quantify changes in strength from the intervention. According to Miyatani et al. [39], the maximum thickness of the *rectus femoris* (RF) and *vastus lateralis* (VL) was obtained at 50% of the distance from the superior and middle tip of the patella to the anterior superior iliac spine. The lateral location of the VL measurement was taken at 10% of the thigh circumference in the lateral direction. With the participant laid supine, the ultrasound probe (12 L linear probe at 10 MHz frequency, gain 80 dB, depth 8 cm) was orientated perpendicular to the muscle fascicles and the skin, with sufficient ultrasound gel to reduce muscle compression. The depth of the image was adjusted until the femur and muscle boundaries were visible on the screen. Three images of each muscle were taken, alternating between muscles, and saved for subsequent analysis. The average of the measures from the three images was used for analysis. The thickness of the VL and RF was defined as the distance from the subcutaneous adipose tissue-muscle interface to either the aponeurosis or the muscle-bone interface. The same expert carried out all ultrasound measurements (CV < 1.8%) and was blinded to which condition each participant was assigned.

#### Blood measurements

The participants attended the laboratory 72 h before the first training session and 72 h after the last training session under fasted conditions for resting blood sample collection in normoxia conditions. In addition, immediately after the first and the last training session at the corresponding environmental condition (S_first_ and S_last_), the antecubital vein of the arm of each participant was catheterized for blood collection. The catheter remained permeable by using a physiological saline solution. Five millilitres of blood were extracted at minutes 5, 10, and 30 post-exercise and poured into tubes with separating gel. We discarded 2 mL of blood before each extraction to avoid dilution of the sample. Blood samples were kept refrigerated at ~ 10 °C and centrifuged in the following 4 h for 10 min at 3000 rpm to separate the serum supernatant, before 500 µl serum aliquots were stored at − 70 °C until analysis. These serum samples were analyzed for growth hormone (GH), testosterone, cortisol, mammalian target of rapamycin (mTOR), interleukin 6 (IL-6), calcium (Ca^2+^), myostatin and microRNA-206. All blood extractions were performed by members of the research team with experience in these measures.

All analyses followed the manufacturer’s instructions. Blood lactate was determined at minutes 5, 10 and 30 of recovery using a Lactate Pro 2 (Arkray, Japan) from the venous blood extracted. Growth hormone, testosterone and cortisol were assessed in a COBAS E-411 System (Roche, Basel, Switzerland) and Ca^2+^ determination was performed in a COBAS C-311 System (Roche, Basel, Switzerland) at minutes 5, 10, and 30 of the recovery. IL-6 was assessed at minutes 5 and 30 of the recovery using the Milliplex Human High Sensitivity T Cell Panel (HSTCMAG-28SK) from Sigma-Aldrich (Darmstadt, Germany). mTOR, phosphor-mTOR (Ser2448) and cell-free total RNA—primarily miRNA—were assessed 30 min post-exercise. mTOR and phosphor-mTOR were measured in a Luminex machine using the Procartaplex™ Multiplex Immunoassay from Thermo Fisher Scientific (Vienna, Austria). Briefly, 25 μL of serum was tested in single replicates in 96-well plates. Each plate contained duplicated serial dilutions (1:4) of a standard sample of known concentration for each analyte provided by the vendor, as well as two blank controls and a reference sample control in duplicate for quality control purposes. Standard curves were used to extrapolate the concentration of the samples, after fitting into a 5-parameter curve algorithm with the LEGENDplex™ Data Analysis Software. The percentage of the active mTOR with respect to the total mTOR was calculated (% active mTOR = phospo mTOR/total mTOR × 100) and used in the analysis. Myostatin was assessed at minutes 5 and 30 of the recovery by ELISA kit (Myostatin [R&D Systems]). Finally, cell-free total RNA—primarily miRNA—was obtained at minute 30 of recovery by miRNeasy Serum/Plasma Kit according to the manufacturer’s instructions (Qiagen, Hilden, Germany). Reverse transcription was performed with a miRCURY LNA RT kit (Qiagen). Quantitative PCR was carried out under standardized conditions with 2 × miRCURY LAN® Master Mix SYBR Green (Qiagen) in a real-time PCR detection system CFX96 (BioRad, California, USA). For relative miRNA quantification, a synthetic non-human miRNA, cel-miR-39, was used as a spike-in control added during RNA extraction. The changes in the fold of the expression of the candidate miRNAs were calculated using the Eq. 2^−ΔΔCt^.

### Statistical analysis

Data are presented as mean ± standard deviation (SD) or mean standard error (SEM). Before analyzing the study’s variables, assumptions of data normality were tested using the Shapiro–Wilk test (*p* > 0.05). Those variables that were not normally distributed were subjected to a transformation process. For blood biomarkers assessed in the S_first_ and S_last_ recovery period when multiple extractions were made, the minimal value (myostatin) or maximal value (all other biomarkers) was taken for statistical analysis.

A one-way ANOVA was used to assess the effect of the environmental condition (N vs. HH vs. NH) on pre- to post-intervention change scores in 1RM and muscle thickness (total [VL + RF]). A two-way repeated measures ANOVA was used to assess the effect of *time* (Δ S_first_ [first session- pretraining] vs. Δ S_last_ [last session—pretraining]), the *environmental condition* (N vs. HH vs. NH), and the interaction between the *time x environmental* condition on GH, testosterone, cortisol, % active mTOR, blood lactate, Ca^2+^, IL-6, and miR-206. Effect sizes through the partial eta-squared (η^2^_p_) value and thresholds (0.02 [small], 0.13 [medium] and 0.26 [large]) were calculated along with ANOVA effects (Bakeman, 2005). Non-normally distributed variables (myostatin) were compared similarly but using the Kruskal–Wallis (within-subjects) or Wilcoxon test (between-subjects) [42]. Effect sizes through the epsilon squared (ε^2^) value and thresholds (0.04 [weak], 0.16 [moderate], 0.36 [relatively strong], 0.64 [strong] and 1.00 [very strong]) were calculated along with Kruskal–Wallis effects. A Bonferroni post hoc test was used to analyze pairwise comparisons.

Cohen’s effect size (ES) was calculated according to the formula d = (M2– M1/SDpooled), where M1 and M2 are the means of the two groups and SDpooled is the pooled standard deviation (*n* is sample size and s^2^ is variance):

$$SDpooled=\sqrt{\frac{\left({n}_{2}- 1\right){s}_{2}^{2} + \left({n}_{1}- 1\right){s}_{1}^{2}}{{n}_{1}+ {n}_{2}- 2}}$$

ES and the mean difference with 90% confidence intervals (CI) were determined for all pairwise comparisons and interpreted as: < 0.20, trivial; 0.20 to 0.59, small; 0.60 to 1.19, moderate; 1.20 to 1.99, large; and > 2.0, very large [26]. All analyses were performed using the software package SPSS (version 26.0, IBM Corp. IBM SPSS Statistics for Windows, Armonk, NY, USA). Effects were considered significant at *p* ≤ 0.10.

## Results

Table 1 shows back squat 1RM and muscle thickness changes from pre-training to week 8 of the R_T_ program. The results for 1RM back squat showed a statistically significant effect of *condition* (*p* = 0.026; η^2^ = 0.215). Although 1RM back squat increased after the R_T_ program in all conditions (*p* < 0.001), NH showed a large significant rise compared to N (ES = 1.20; *p* = 0.023). Muscle thickness measures of RF and VL showed a significant *condition* effect (*p* < 0.054; η^2^ > 0.178). Muscle growth similarly improved in N and HH groups after the R_T_ program (ES <  − 0.15; *p* = 1.0), while NH did not display any change (*p* > 0.10).

**Table 1:** Condition training effect on the absolute change in the maximal strength and muscle thickness

	Condition	N	HH	NH	*p*-value [ES] Adjusted between-group differences [90% CI]		
		Mean ± SD	Mean ± SD	Mean ± SD			
1RM back squat (kg)	**Baseline**	95.9 ± 14.7	80.5 ± 14.8	96.6 ± 18.6	**ΔHH vs. N**	**ΔNH vs. N**	**ΔHH vs. NH**
	**Post–Pre**	16.0 ± 7.9***	23.3 ± 9.2***	27.2 ± 10.2***	0.272 [0.85] 7.24 [− 1.99; 16.47]	**0.023** [1.20] 11.12 [2.44; 19.81]	0.979 [-0.40] − 3.89 [− 12.57; 4.80]
					***F***_***2,30***_ = *4.119*; ***p*** = *0.026;* ***η***^***2***^ = *0.215*		
Muscle thickness (VL + RF) (mm)	**Baseline**	5.23 ± 0.65	5.54 ± 0.71	5.47 ± 0.41	**ΔHH vs. N**	**ΔNH vs. N**	**ΔHH vs. NH**
	**Post–Pre**	0.46 ± 0.31**	0.41 ± 0.27***	0.10 ± 0.24	1.000 [− 0.14] − 0.04 [− 0.31; 0.30]	**0.012** [− 1.30] − 0.35 [− 0.61; − 0.1]	**0.031** [1.22] 0.31 [0.06; 0.57]
					***F***_***2,30***_ = *6.125;* ***p*** = *0.006;* ***η***^***2***^ = *0.286*		

*1RMSQ*, 1 repetition maximum on squat; *VL*, vastus lateralis; RF: rectus femoris; *N*, normoxia; *HH*, hypobaric hypoxia; *NH*, normobaric hypoxia; *Post–pre*, post-training value at 8 weeks – pre-training; *SD*, standard deviation; *p*, p value for the statistical test (one-way ANOVA); *η*^*2*^, eta square; *F*, F test; Adjusted between-group difference is the estimated marginal mean of the difference between the environmental condition (HH vs. N; NH vs. N; HH vs. NH) after adjusting for baseline differences; p-value of the adjusted between-group difference. * Differences with respect pre-value in N, HH and NH (*** *p* < 0.001; ** *p* < 0.05; * *p* < 0.10)

The serum GH, testosterone, cortisol and % active mTOR changes of the adjusted to pre-training peak value in the first (S_first_) and the last session (S_last_) of the R_T_ program are presented in Table 2. Post-exercise GH increased in all conditions and sessions monitored (*p* < 0.001). Results in GH displayed a *time* (*p* = 0.037; η^2^_p_ = 0.137) and a *time x condition* interaction (*p* = 0.043; η^2^_p_ = 0.189) effect. R_T_ maintained or discreetly reduced the release of this hormone in all groups. HH group showed a moderate to large diminished value in GH at the beginning of the program (S_first_) compared to N and NH; significance was only reached with NH (ES =  − 1.30; *p* = 0.086). Testosterone did not change from the pre-exercise throughout the training program in all groups (*p* > 0.10), although a slightly elevated value was observed at the end of the program in both hypoxia conditions (ES > 0.95). Substantial interindividual variability in the cortisol response makes interpretation difficult. The analysis only detected an elevated response in cortisol in HH at the beginning of the program (*p* < 0.05). Finally, the % active mTOR exhibited a significant effect of *time* (*p* = 0.053; η^2^_p_ = 0.119), *condition* (*p* = 0.001; η^2^_p_ = 0.354) and a *time x condition* interaction (*p* = 0.039; η^2^_p_ = 0.195). HH and N groups tend to reduce the % active mTOR throughout the program, although the HH condition displayed values above pre-exercise (ES > 0.33; *p* < 0.10). The NH group did not show changes of interest in this variable.

**Table 2:** Adjusted standardized mean differences between conditions of hormones and %active mTOR

	Condition	N	HH	NH	*p*-value [ES] Adjusted between-group differences [90% CI]					
	Session	Mean ± SEM	Mean ± SEM	Mean ± SEM						
GH (pg/mL)	**Baseline**	269 ± 78.1	1131 ± 544	412 ± 138	**ΔHH vs. N**			**ΔNH vs. N**	**ΔHH vs. NH**	
	**ΔS**_**first**_	15,165 ± 4460**	7584 ± 1743***	17,113 ± 2332***	0.287 [− 0.71] − 7581.5 [− 17,409.9; 2246.9]			1.000 [0.17] 1947.6 [− 7296.4; 11,191.6]	**0.086** [− 1.30] − 9529.1 [− 18,773.1; − 285.2]	
	**ΔS**_**last**_	11,221 ± 2414**	9403 ± 1681***	9464 ± 1223***	1.000 [− 0.28] − 1817.9 [− 7613.0; 3977.1]			1.000 [− 0.29] − 1756.9 [− 7207.3; 3693]	1.000 [− 0.01] − 61.1 [− 5511.6; 5389.4]	
					***Time effect:*** *F*_*1,30*_ = *4.750;* *p* = *0.037;* *η*^*2*^_*p*_ = *0.137* ***Condition effect:*** *F*_*2,30*_ = *1.667;* *p* = *0.206;* *η*^*2*^_*p*_ = *0.100* ***Time × condition effect:*** *F*_*2,30*_ = *3.496;* *p* = *0.043;* *η*^*2*^_*p*_ = *0.189*					
Testosterone (ng/mL)	**Baseline**	7.27 ± 0.71	5.73 ± 0.32	6.28 ± 0.31	**ΔHH vs. N**		**ΔNH vs. N**			**ΔHH vs. NH**
	**ΔS**_**first**_	0.29 ± 0.33	0.34 ± 0.39	0.44 ± 0.58	1.000 [0.05] 0.05 [− 1.53; 1.64]		1.000 [0.09] 0.15 [− 1.34; 1.64]			1.000 [− 0.06] 0.19 [− 1.16; 1.54]
	**ΔS**_**last**_	− 0.94 ± 0.58	0.77 ± 0.30	0.58 ± 0.39	0.037 [1.17] 1.71 [0.27; 3.15]		**0.053** [0.95] 1.52 [0.17; 2.87]			1.000 [0.16] 0.19 [− 1.16; 1.54]
					*** Time effect:*** *F*_*1,30*_ = *0.375;* *p* = *0.545;* *η*^*2*^_*p*_ = *0.012* ***Condition effect:*** *F*_*2,30*_ = *2.002;* *p* = *0.153;* *η*^*2*^_*p*_ = *0.118* *** Time × condition effect:*** *F*_*2,30*_ = *1.969;* *p* = *0.157;* *η*^*2*^_*p*_= *0.116*					
Cortisol (mmol/L)	**Baseline**	574 ± 27.3	439 ± 38.6	495 ± 25.1	**ΔHH vs. N**	**ΔNH vs. N**				**ΔHH vs. NH**
	**ΔS**_**first**_	4.36 ± 53.8	130 ± 63.9**	106 ± 65.3	0.555 [0.68] 125.94 [− 81.15; 333.03]	0.755 [0.49] 102.09 [− 92.69; 296.87]				1.000 [0.11] 23.85 [− 170.93; 218.63]
	**ΔS**_**last**_	102 ± 53.5	123 ± 55.4	− 65.2 ± 50.7	1.000 [0.12] 20.55 [− 155.64; 196.73]	**0.095** [− 0.95] − 167.57 [− 333.28; − 1.86]				**0.050** [1.05] 188.11 [22.40; 353.82]
					***Time effect: ****F*_*2,30*_ = *0.977;* *p* = *0.331;* *η*^*2*^_*p*_ = *0.032* ***Condition effect: ****F*_*2,30*_ = *1.041;* *p* = *0.366;* *η*^*2*^_*p*_ = *0.065* ***Time × condition effect: ***F_2,30_ = 8.817; *p* = 0.001; η^2^_p_ = 0.370					
% active mTOR	**Baseline**	68.3 ± 3.19	90.6 ± 8.66	100 ± 3.69	**ΔHH vs. N**	**ΔNH vs. N**				**ΔHH vs. NH**
	**ΔS**_**first**_	23.8 ± 9.5*	147 ± 52.2**	0.95 ± 4.8	0.017 [1.04] 123.55 [31.17; 215.94]	1.000 [− 0.97] − 22.89 [− 109.78; 64.00]				0.002 [1.34] 146.44 [59.55; 233.33]
	**ΔS**_**last**_	13.9 ± 13.7	36.6 ± 27.8*	6.7 ± 6.4	1.000 [0.33] 22.73 [-32.72; 78.18]	1.000 [− 0.22] − 7.16 [− 59.32; 45.00]				0.633 [0.50] 29.89 [− 22.27; 82.05]
					*** Time effect:*** *F*_*1,30*_ = *4.07;* *p* = *0.053;* *η*^*2*^_*p*_ = *0.119* ***Condition effect:*** *F*_*2,30*_ = *8.24*; *p* = *0.001;* *η*^*2*^_*p*_ = *0.354* ***Time × condition effect:*** *F*_*2,30*_ = *3.63;* *p* = *0.039;* *η*^*2*^_*p*_ = *0.195*					

*GH*, growth hormone; *mTOR*, mammalian target of rapamycin; *N*, normoxia; *HH*, hypobaric hypoxia; *NH*, normobaric hypoxia; *ΔS*_*first*_, first session of the training program – pre-training; *ΔS*_*last*_, last session of the training program – pre-training; *SEM*, standard error of the mean; *p*, p value for the statistical test (ANOVA); *η*^*2*^_*p*_, partial eta square; *F*, F test; Adjusted between-group difference is the estimated marginal mean of the difference between the environmental condition (HH vs. N; NH vs. N; HH vs. NH) at first and last session of training after adjusting for baseline differences; *p*-value of the adjusted between-group difference. * Differences with respect to pre-exercise in N, HH and NH (*** *p* < 0.001; ** *p* < 0.05; * *p* < 0.10)

Adjusted pre-exercise mean change of IL-6, miR-206 and myostatin in S_first_ and S_last_ are displayed in Table 3. The IL-6 showed a significant effect of *time* (*p* = 0.060; η^2^_p_ = 0.113), *condition* (*p* = 0.004; η^2^_p_ = 0.305) and a *time x condition* interaction (*p* = 0.019; η^2^_p_ = 0.231). Compared to N, the HH and NH groups increased IL-6 values in S_first_ (ES > 1.12; *p* < 0.011), while S_last_ displayed a moderate reduction towards pre-exercise values in all groups. IL-6 displayed the highest values in NH throughout the study, reaching a large difference with HH in S_last_ (ES = -1.17; *p* = 0.024). Circulating miR-206 revealed a significant effect of the *condition* (*p* = 0.026; η^2^_p_ = 0.215) and a *time x condition* interaction (*p* = 0.009; η^2^_p_ = 0.272). Despite not detecting a *time* effect, a moderate to large increase in serum miR-206 was observed from S_first_ to S_last_ in N (ES = 0.76) and HH (ES = 1.65). Compared to N and HH, the NH group displayed the highest serum miR-206 values throughout the program (ES ranged from − 1.48 to 0.33). Contrarily, myostatin serum values presented a clear tendency to reduce its value in N and HH while increasing in NH at the end of the training period (ES > [1.09]).

**Table 3:** Adjusted standardized mean differences between conditions of IL-6, miR-206 and myostatin

	Condition	N	HH	NH	*p*-value [ES] Adjusted between-group differences [90% CI]			
	Session	Mean ± SEM	Mean ± SEM	Mean ± SEM				
IL-6 (pg/mL)	**Baseline**	45.7 ± 18.2	50.8 ± 19.1	10.1 ± 3.09	**ΔHH vs. N**	**ΔNH vs. N**		**ΔHH vs. NH**
	**ΔS**_**first**_	− 19.1 ± 7.33**	5.92 ± 6.84	8.18 ± 1.60***	**0.011** [1.12] 25.01 [7.31; 42.72]	**0.003** [1.73] 27.27 [10.62; 43.92]		1.000 [− 0.15] − 2.26 [− 18.91; 14.40]
	**ΔS**_**last**_	− 13.2 ± 6.45*	− 21.8 ± 9.78*	3.54 ± 2.79	1.000 [− 0.33] − 8.64 [− 29.85; 12.58]	0.213 [1.09] 16.75 [− 3.20; 36.71]		**0.024** [− 1.17] − 25.39 [− 45.34; − 5.44]
					***Time effect:*** *F*_*1,30*_ = *3.829;* *p* = *0.060;* *η*^*2*^_*p*_ = *0.113* ***Condition effect:*** *F*_*1,30*_ = *6.576;* *p* = *0.004;* *η*^*2*^_*p*_ = *0.305* ***Time × condition effect:*** *F*_*2,30*_ = *4.503;* *p* = *0.019;* *η*^*2*^_*p*_ = *0.231*			
miR-206 (2E-ΔΔCq)	**Baseline**	0.76 ± 0.13	0.91 ± 0.15	1.43 ± 0.27	**ΔHH vs. N**	**ΔNH vs. N**		**ΔHH vs. NH**
	**ΔS**_**first**_	0.15 ± 0.17	− 0.61 ± 0.09***	1.98 ± 0.64**	0.794 [− 1.80] − 0.76 [− 2.25; 0.73]	**0.020** [1.03] 1.84 [0.43; 3.24]		**0.001** [− 1.48] − 2.60 [− 3.99; − 1.19]
	**ΔS**_**last**_	0.62 ± 0.22**	0.84 ± 0.38*	1.2 ± 0.62*	1.000 [0.22] 0.22 [− 1.39; 1.82]	1.000 [0.33] 0.58 [− 0.93; 2.09]		1.000 [− 0.19] − 0.36 [− 1.87; 1.15]
					***Time effect:*** *F*_*1,30*_ = *1.829;* *p* = *0.186;* *η*^*2*^_*p*_ = *0.057* ***Condition effect:*** *F*_*2,30*_ = *4.120;* *p* = *0.026;* *η*^*2*^_*p*_ = *0.215* ***Time × condition effect:*** *F*_*2,30*_ = *5.607;* *p* = *0.009;* *η*^*2*^_*p*_ = *0.272*			
Myostatin (pg/mL)	**Baseline**	2118 ± 772	2911 ± 837	2639 ± 880	**ΔHH vs. N**		**ΔNH vs. N**	**ΔHH vs. NH**
	**ΔS**_**first**_	1513 ± 821	969 ± 552*	− 297 ± 220**	1.000 [1.12] − 544.22 [− 2324.91; 1236.48]		**0.089** [1.73] − 1809.76 [− 3484.58; − 134.95]	**0.031** [− 0.15] 1265.55 [− 409.27; 2940.37]
	**ΔS**_**last**_	756 ± 444	785 ± 780	7511 ± 2933**	1.000 [− 0.33] 29.58 [− 6820.33; 6879.50]		0.366 [1.09] 6755.33 [312.71; 13,197.94]	0.442 [− 1.17] − 6725.75 [− 13.168.37; − 283.13]
					***Condition effect S1:*** *χ*^*2*^ = *7.98;* *p* = *0.019;* *ε*^*2*^ = *0.249* ***Condition effect S22:*** *χ*^*2*^ = *3.14;* *p* = *0.208;* *ε*^*2*^ = *0.098* ***Time effect:*** *W* = *382;* *p* = *0.071*			

*IL-6*, interleukin 6; *miR-206*, microRNA-206; *N*, normoxia; *HH*, hypobaric hypoxia; *NH*, normobaric hypoxia; *ΔS*_*first*_, first session of the training program – pre-training; *ΔS*_*last*_, last session of the training program – pre-training; *SEM*, standard error of the mean; *p*, p value for the statistical test (ANOVA); *η*^*2*^_*p*_, partial eta square; *F*, F test; χ2, chi-square; ε2, epsilon squared; W, Wilcoxon test;  Adjusted between-group difference is the estimated marginal mean of the difference between the environmental condition (HH vs. N; NH vs. N; HH vs. NH) at first and last session of training after adjusting for baseline differences; *p*-value of the adjusted between-group difference. * Differences with respect to pre-exercise in N, HH and NH (*** p < 0.001; ** *p* < 0.05; * *p* < 0.10)

Adjusted to pre-exercise mean difference between peak values of Ca^2+^ and blood lactate in S_first_ and S_last_ are shown in Table 4. Analysis showed only a significant effect of *condition* (*p* < 0.011; η^2^_p_ > 0.259) for Ca^2+^ and lactate. Pairs comparison for Ca^2+^ presented low values in NH when compared to HH (ES > 1.27; *p* < 0.055) in both testing sessions. Maximal blood lactate concentration showed similar values between HH and N, both larger over NH; significance was only reached with N (ES <  − 1.19; *p* < 0.032).

**Table 4:** Adjusted standardized mean differences between conditions of metabolites

	Condition	N	HH	NH	*p*-value [ES] Adjusted between-group differences [90% CI]		
	Weeks	Mean ± SD	Mean ± SD	Mean ± SD			
Ca^2+^ (mmol/L)	**Baseline**	2.34 ± 0.06	2.36 ± 0.05	2.36 ± 0.05	**ΔHH vs. N**	**ΔNH vs. N**	**ΔHH vs. NH**
	**ΔS**_**first**_	0.14 ± 0.10**	0.15 ± 0.06***	0.05 ± 0.07**	1.000 [0.10] 0.01 [− 0.07; 0.09]	**0.025** [− 1.09] − 0.10 [− 0.17; -0.02]	**0.013** [1.54] 0.10 [0.03; 0.18]
	**ΔS**_**last**_	0.11 ± 0.07***	0.13 ± 0.04***	0.07 ± 0.05***	1.000 [0.23] 0.01 [− 0.04; 0.07]	0.191 [− 0.73] − 0.04 [− 0.09; 0.01]	**0.055** [1.27] 0.06 [0.01; 0.10]
					***Time effect: ****F*_*1,30*_ = *0.397;* *p* = *0.533;* *η*^*2*^_*p*_ = *0.013* *** Condition effect:*** *F*_*2,30*_ = *6.307;* *p* = *0.005;* *η*^*2*^_*p*_ = *0.296* ***Time × condition effect:*** *F*_*2,30*_ = *2.256;* *p* = *0.122;* *η*^*2*^_*p*_ = *0.131*		
Blood Lactate (mmol/L)	**Baseline**	1.99 ± 0.47	2.63 ± 0.68	1.96 ± 0.37	**ΔHH vs. N**	**ΔNH vs. N**	**ΔHH vs. NH**
	**ΔS**_**first**_	14.9 ± 3.51***	13.0 ± 3.14***	11.4 ± 2.46***	0.546 [− 0.55] − 1.84 [− 4.84; 1.16]	**0.029** [− 1.19] − 3.50 [− 6.33; − 0.68]	0.599 [0.60] 1.66 [− 1.16; 4.49]
	**ΔS**_**last**_	14.8 ± 2.12***	12.9 ± 3.03***	11.8 ± 2.66***	0.329 [− 0.74] − 1.94 [− 4.57; 0.69]	**0.032** [− 1.23] − 3.01 [− 5.48; − 0.54]	1.000 [0.38] 1.07 [− 1.40; 3.54]
					***Time effect: ****F*_*1,30*_ = *0.018;* *p* = *0.894;* *η*^*2*^_*p*_ = *0.001* ***Condition effect:*** *F*_*2,30*_ = *6.307;* *p* = *0.005;* *η*^*2*^_*p*_ = *0.296* ***Time × condition effect:*** *F*_*2,30*_ = *2.256;* *p* = *0.122;* *η*^*2*^_*p*_ = *0.131*		

*Ca*^*2*+^, Calcium; *N*, normoxia; *HH*, hypobaric hypoxia; *NH*, normobaric hypoxia; *ΔS*_*first*_, first session of the training program – pre-training; *ΔS*_*last*_, last session of the training program – pre-training; *SD*, standard deviation; *p*, p value for the statistical test (ANOVA); *η*^*2*^_*p*_, partial eta square; *F*, F test; Adjusted between-group difference is the estimated marginal mean of the difference between the environmental condition (HH vs. N; NH vs. N; HH vs. NH) at first and last session of training after adjusting for baseline differences; p-value of the adjusted between-group difference. * Differences with respect to pre-exercise in N, HH and NH (*** *p* < 0.001; ** *p* < 0.05; * *p* < 0.10)

## Discussion

This study aimed to analyze the effect of an 8-week R_T_ period at terrestrial and normobaric hypoxia on both muscle hypertrophy and maximal strength development with respect to the same training in normoxia. Although all groups improved 1RM, the main findings reveal that the hypoxia condition, especially NH, benefited the most. The highest increase in % active mTOR was shown in the HH group, while NH displayed the more pronounced inflammatory response and circulating miR-206, both linked to muscle growth, throughout the training period. In contrast, elevated myostatin was observed in NH, along with gains in muscle thickness favoring N and HH groups over NH, which does not support the expected additional benefit of RTH over RTN on muscle growth. Therefore, different responses to training were found between terrestrial and normobaric hypoxia, suggesting the NH condition as the least favorable for muscle growth, although it seemed to benefit strength development.

Several previous studies have shown a period of RTH to enhance muscle growth and strength [28, 29, 32]. In contrast, the results of this study show an increase in muscle thickness of the lower limbs in N (8.03%) and HH (5.49%), while a significant improvement was not observed in the NH condition (1.83%) (Table 1). The benefit of hypoxia on muscle hypertrophy is possibly associated with higher metabolic stress during exercise [51], and the upregulation of the inflammatory adaptative response [6, 8]. Moreover, the absence of clear differences between environmental conditions in Ca^2+^ response and the maintenance of the highest serum IL-6 values in NH contrasts again with the small increase in muscle gains recorded in this group. Therefore, the time-course exploration of the inflammatory and metabolic stress variables and their relationship with muscle adaptations in both hypoxia types needs further exploration. Although the NH group experienced no significant improvement in muscle growth, this condition displayed the largest increase in back squat 1RM after the training (Table 1). Some research has observed RTH to improve strength regardless of muscle structure changes [7, 15, 19, 23, 43], likely due to neuromuscular adaptations. This is supported by a recent study, which demonstrated a small effect (ES = 0.38) for acutely increased excitability of the most excitable structures of the corticospinal tract (5.3% lower resting motor threshold) when exposed to 2320 m asl compared to normoxic conditions [35]. Unfortunately, conclusions about neuromuscular adaptations to R_T_ are not possible from this study, as neuromuscular variables were not examined. Moreover, all the available literature on the topic was conducted under NH, and to our knowledge this is the first RTH study including both terrestrial and normobaric hypoxia for comparative purposes.

The R_T_ period showed elevated circulating GH in all groups, although testosterone remained near pre-training values. Consistent with previous research [6, 20], the increase in GH at S_first_ was slightly blunted in HH in S_first_ compared to N and NH. This could be partially explained by the bigger sympathetic stimulation related to terrestrial hypoxia, optimizing the ventilatory response [44] and improving the buffer capacity system [5]. Consistent with this hypothesis, cortisol dynamics should have reflected the global stress and metabolic requirements of exercise [53]. Accordingly, HH presents an elevation of circulating cortisol above pre-exercise values at the beginning of the program. This result, joined with the decrease in the stress response expected at the end of the training period, could justify the *time x condition* interaction detected in this variable. Otherwise, circulating hormones were similar between conditions at the end of the R_T_ program, showing a good adaptive response to exercise training (see Table 2). The literature on hormonal responses to RTH is inconsistent, highlighting the complexity of the hormonal response to this exercise modality.

Moreover, the results displayed a % active mTOR largely upregulated at the beginning of the R_T_ period in the HH condition, although this difference concerning N and NH seems not to be enough to discriminate the net muscle mass gains between groups at the end of the training period. There were no reference data from a recovery period similar to the one used in this study. Other research demonstrated a downregulation [18, 47] or unaltered values 3 h after R_T_ exercise in severe simulated hypoxia [17]. Nevertheless, the role and contribution of single measurements of mTOR remain speculative due to the multiple targets and mechanisms involved in the signaling processes [31].

Overexpression of miR-206 has been associated with muscle differentiation, activation of satellite cells, myogenesis and/or protein synthesis promotion [16, 33, 52] while myostatin operates as a negative regulator of the major protein synthetic pathways in skeletal muscle [25, 30]. In accordance with other research, the beginning of the training period that displayed a large increment of circulating miR-206 in NH [13], which joined with the decrease of myostatin [3, 41], may favor muscle cell differentiation and regeneration [16, 33]. However, at the end of the training period, the overexpression of miR-206 and the increase in myostatin levels exhibited in NH could negatively affect muscular satellite cell proliferation and differentiation, although pair comparisons did not find significant differences between conditions. The behavior of these variables could promote non-hypertrophy-directed changes, but instead, a potentiated muscle repair-oriented process [11]. Moreover, the air quality factors linked to NH equipment with a low flow rate could elicit an additional stimulus to the individual response to hypoxia [54] and the R_T_ program. Further research is required to investigate this hypothesis. Moreover, the miRNA response could be conditioned by other factors, such as the type of muscle fiber predominance [61] or the variability in response to hypertrophy training (low or high responders) [14].

This research has some limitations that should be noted. Firstly, the sample size was relatively small, which could influence the width of probability distributions across the outcomes. Logistical constraints and cost of biomarker analyses limited a larger sample being recruited. Nevertheless, results from this population are of specific interest. Secondly, it is possible that some air quality factors in the NH condition, in addition to the reduced FiO_2_, affected the net stress on participants. The NH tent used provides a small space (11.5 m^3^), which can result in substantial accumulation of carbon dioxide, high temperature and relative humidity during high-intensity exercise [58], affecting the SpO_2_ for the same FiO_2_ [4, 59]. While speculative, this may have created conditions for the NH group which were not conducive to muscle hypertrophy.

## Conclusions

In conclusion, the findings from this study suggest that 8 weeks of RTH does not augment muscle growth, although it may enhance strength gains. The greater muscle growth achieved in HH over NH confirms differences between both types of hypoxia, which is also reflected in the acute and chronic responses of some biomarkers, such as miR-206 and myostatin, without finding differences between hypoxia types on muscle strength. Some variables related to air quality and linked to the NH equipment used in this study could affect the net stress of the NH group. Future studies should include a description of the dimensions, flow rate capacity of the hypoxia system and air quality, beyond the FiO_2_, to control potential stressors’ impact on the outcomes when using NH equipment.

## Data Availability

All data supporting the findings are available from the corresponding author upon reasonable request.
